# Aids to Nursing

**Published:** 1888-05-12

**Authors:** M. M. Basil

**Affiliations:** Author of "The Commoner Accidents to Life and Limb."


### Aids to Nursing.

By M. M. Basil, M.A., M.B., C.M., Author of "The Commoner Accidents to Life and Limb."

*(Continued from p. 57.)*

BATHING AND BATHS—*continued.*

Sometimes cold water is used to reduce the temperature of the body in high fever. It would not be advisable for a nurse to undertake to conduct this process, even when she has seen it done time after time. It can no more be done with safety from the experience got by seeing others do it than an amputation at the hip joint or an abdominal section can be done by merely seeing these operations performed during your hospital training.

A cold bath usually lasts from three to five minutes, a warm one fifteen to twenty minutes; the time, however, for a warm bath varies much according to circumstances.

The *hip-bath* is the commonest of the *partial* baths. When used to soothe pain or aching of the parts its temperature is about 98° to 100° F.; in abnormal conditions of the pelvic organs, however, a temperature of 110° to 115°F. must be employed for fifteen to twenty minutes. The patient's body and extremities should be well protected against chills.

Hot-air baths, vapour baths, the wet pack, etc., require no special description, as they should always be given under careful supervision, and their use is best learnt by experience in the wards.

In giving the hot-air bath you should never leave the patient alone even for a second; for, should the clothes catch fire accidentally in your absence, the patient will be placed in a terrible position. The lamp should be withdrawn as soon as perspiration begins to break out. If the patient does not perspire, you may sponge him all over with some water and try the process again without previously rubbing him dry. Always be prepared for the immediate treatment of sudden fainting before giving a vapour bath. It must be borne in mind that the kidneys are often stimulated to action in the wet pack. Steps should therefore be taken before wrapping the sheets to prevent the discomfort which is certain to arise in the absence of conveniences for relieving the bladder. The wet pack is kept on for an hour or less. Dr. Cullingworth mentions three to four hours; that, however, must be under exceptional conditions.

Baths may be medicated in various ways. It is quite unnecessary to burden your memory with any figures in this connexion, but for purposes of reference it is best to group them here together:

1. Soda bath: 1 lb. of carbonate of soda, *i.e.*, washing soda, to the bath, *i.e.*, 30 gallons of water.
2. Sulphur bath: ¼ lb. of sulphuret of potassium to the bath. Use a *wooden* vessel.
3. Salt water bath: ½ lb. salt to 4 gallons of water.
4. Mustard foot-bath: oz. ij. to iv. to 4 gallons of hot water, or 3 to 4 table-spoonfuls to the foot-bath.
5. Bran bath: boil 1 lb. of bran for 15 minutes, strain, and then add it to the bath.
6. Starch bath: starch or potato-mash ½ lb., to 2 to 3 quarts of water.
7. Gelatine bath: ¼ lb. to ½ lb. of gelatine to quart of water.
8. Nitro-muriatic acid bath (Martin's): concentrated muriatic acid, pure, 3 parts by measure; strong nitric acid, 2 parts. Mix the two acids slowly and carefully; after 20 minutes, add distilled water 5 parts; mix well. For a foot or sponging bath, 6oz. of the above mixture is to be added to 2 gallons of water at 98°F., and well mixed.

POULTICES, FOMENTATIONS, SINAPISMS, LOTIONS, &c.

We shall take up next the examination of the various local applications. The choice of the particular remedy for each case will be made for you by the doctor, but to carry out his instructions intelligently, you must understand a few principles applicable to the whole group of these remedies. In the first place, a warm or hot application must be kept warm from beginning to the end. If applied in an interrupted manner, no good, but positive harm, will be done to the patient. If, for example, a poultice which is hot the first hour is allowed to remain in contact with the part another hour when cold, and then the surface of the body is exposed to the air while the next poultice is being got ready, the patient will not merely derive no benefit from the treatment, but his disease will positively be made worse by the remedy. The same consideration applies with far greater strictness to cold or cooling applications. Continuous cold will, under certain conditions, subdue inflammation; interrupted cold will bring on inflammation where it does not exist, and aggravate it where it is already present. If ice-bags and evaporating lotions cannot be applied persistently, it is far better to give up all attempts at using them, than to add to the disease by their interrupted application. To keep your cold applications cold, your warm ones warm, must be observed as your rule from first to last.

Then, again, all evaporating lotions are prescribed with the object of their acting as cold applications. A very little difference, however, in the method of their management will convert them into hot and irritating agents. It is quite possible to produce exactly opposite results with one and the same remedy according to the method of application; it therefore behoves every nurse to clearly understand the object which is to be secured in each case, and the method in which the application must be made to attain that object. Nearly all evaporating lotions contain alcohol in one form or another. Now, alcohol applied to the healthy or inflamed skin will either cool and soothe it or irritate and inflame it, according as it is allowed to be converted into vapour freely and leave the surface, or prevented from doing so. Sulphuric ether and chloroform are other familiar liquids which act in the same manner, and you can make an instructive experiment on yourselves any day. If a little ether be poured on the hand and exposed to the air it soon disappears, and a cooling sensation is produced. On the other hand, if you wet two or more folds of lint with ether, apply it to the body, and then put some impermeable material over it, so as to keep the ether-vapour in contact with the skin, you will find that it reddens and irritates the part.

This cooling effect of alcohol, when allowed to pass into vapour, is taken advantage of in all evaporating lotions to produce definite results. If, however, instead of being applied on a thin fold of lint, the lotion is applied on several folds, and then covered with cotton-wool or other protective, it no longer acts as a cooling agent, but a counter-irritant.

Poultices of various forms are amongst the most common of the local applications—linseed meal, bread, starch, soap and sugar, oakum, and figs are all used, according to circumstances. They may be applied on tow, soft linen rag, or, best of all, where cost is no consideration, on muslin.

Of these, linseed poultice alone will be considered in detail here.

The meal must be obtained from the crushed seeds, without any previous extraction of the oil. When pure, it is not gritty to the feel, and a pinch of it thrown on water does not sink rapidly to the bottom. It is important to get good linseed meal, as sand and other articles used for adulterating it are sure to irritate the skin, and produce a crop of painful pimples.

Have everything ready before you begin to make a poultice. The portion of body where it is to be applied must be made antiseptic and clean, and the material on which the poultice is to be applied must be warmed. To make a linseed poultice it is essential that the water is not merely hot, but actually boiling. Water which has been boiled at some indefinite time will not do. No form of the past tense will answer here.

Scald out the basin just before use; pour the meal gradually into the boiling water, not *vice versa*. With a spatula or stout spoon you then stir up the mixture while the meal is being poured in until you have it of the proper consistency, and spread it rapidly and evenly. To effect this with dispatch you must moisten the spatula from time to time with boiling water. A properly made poultice must be hot, moist, and even—not a knotty, lumpy dab. It must not be made before the patient is ready, allowed to get cold, and then heated near a fire. It must be held close to the face to ascertain its temperature just before application. As a rule, the hand is not sensitive enough for this. A little pure olive oil or vaseline smeared on the surface is often of advantage. It must be allowed to cover the part gradually, not clapped on all at once. In nervous people it is sometimes necessary to place the hand between it and the skin, and then to withdraw it gradually, so as to diminish the shock from fright.

The size of the poultice will depend on circumstances. The thickness of it, too, may vary much—one-half to one inch or more; in rare cases one-eighth to one-quarter of an inch. The edges must be nearly as thick as the centre. A poultice must be changed as soon as it is felt to be cold. This is best determined by introducing the finger, from time to time, between it and the skin, after the first hour of its application. All average statements on the point are valueless, if not mischievous, as there are so many modifying circumstances to be taken into account. Hence we have variations from "two, or at most three hours" (Dr. Ringer), to "six to eight hours" (Dr. Cullingworth).

Poultices can be kept warm by covering them with spongiopiline, mackintosh, or cotton wool, or a combination of the last two. Before taking a poultice off, have the next one ready to take its place.

What should we have under a poultice? Should there be anything at all interposed between it and the skin? "Linseed meal poultices," says one authority, "are always placed next to the skin, and should never have a covering of muslin or anything else between." "There should be some muslin," says another, "under a poultice; some mackintosh over it." There is no single principle applicable to all cases, and the best course for you is to inquire from the doctor when in doubt in any case. The general considerations which may guide you are:

1. In many children, and a few nervous women and men, to interpose some muslin or soft flannel, to diminish the shock from a large poultice. No one can have nursed children long without observing that a poultice is not always a trifling matter to a child. The idea that muslin or flannel interferes with the action of a poultice is mainly a fanciful one.

2. In surgical wards where adults are treated, apply a poultice nearly always directly to the body.

3. In medical cases it is frequently of great advantage to interpose some material between the skin and the poultice. Where deep-seated pain is to be relieved—as, for example, in cases of abdominal or thoracic disease—it will often be possible by such means to use a poultice which is extremely hot, and succeed in relieving painful conditions on which an ordinary poultice has had no effect. Hence one of the most efficient means of making a jacket poultice for cases of pneumonia is to arrange a bag whose mouth is the upper end of the length of the "jacket." Very hot meal can be poured into this, and kept in position by tapes arranged on the principle of braces. Poultices are often inefficient in medical cases, because most nurses learn poultice-making in the surgical wards, and carry the lesson to the medical side without necessary modifications.

Linseed poultices have an objectionable smell. They also at times irritate the skin and produce a crop of pimples This irritation can frequently be prevented by previous washing of the skin with soap and water, sulphuric ether, or turpentine, so as to remove all oily excretions; by subsequent cleanliness each time the poultice is changed; by the use of pure meal, and of vaseline in place of any vegetable or animal oil. Should a crop of pimples appear, the skin around must be protected by plasters in the centre of which a hole has been cut out for the application of poultices, or linseed poultices must be discontinued and starch or oatmeal ones substituted.

In a hospital it is convenient to have the means of making poultices in large numbers with rapidity. It would be best, however, for nurses to learn to depend at times only on such requisites as can be found in most houses. The formidable farrago of requisites mentioned in some of the books for making what they choose to call a "proper poultice," cannot be had outside a hospital, and the majority of people must be satisfied with poultices that are *not* proper.

*(To be continued.)*

